# Muscle Wasting in Diabetes: A Case Report Evaluating a Rare Neuropathic Complication of Diabetic Microvasculitis

**DOI:** 10.7759/cureus.106382

**Published:** 2026-04-03

**Authors:** Tiffany T Nguyen, Shriya Deshpande, Kelly Huynh, Norman Chow

**Affiliations:** 1 Physical Medicine and Rehabilitation, California Health Sciences University, Clovis, USA; 2 Neurology, California Health Sciences University, Clovis, USA; 3 Family Medicine, California Health Sciences University, Clovis, USA; 4 Family Medicine, Blossom Hill Medical Group, Los Gatos, USA

**Keywords:** diabetes type 2, diabetic amyotrophy, diabetic lumbosacral radiculoplexus neuropathy, diagnostic criteria, lumbar radiculopathy

## Abstract

Proximal lower extremity weakness in patients with diabetes mellitus presents a diagnostic challenge due to overlapping etiologies, including diabetic amyotrophy, lumbar radiculopathy, and chronic inflammatory demyelinating polyneuropathy (CIDP). This report presents the case of an 80-year-old male with a history of type 2 diabetes mellitus who experienced recurrent falls, progressive right thigh weakness, and muscle atrophy. Although the clinical presentation initially suggested diabetic amyotrophy, electrodiagnostic studies and imaging failed to support this diagnosis. Instead, findings were more consistent with peripheral neuropathy and possible focal L5 radiculopathy. This case highlights the limitations of current diagnostic frameworks for diabetic amyotrophy, particularly in atypical presentations that lack classic features such as pain and characteristic electromyographic abnormalities. The absence of standardized diagnostic criteria necessitated a diagnosis of exclusion, emphasizing the importance of clinical judgment. This report underscores a structured approach to evaluating diabetic patients with lower extremity weakness, including the role of a comprehensive workup, the exclusion of diagnoses with similar presentations, and the potential for recovery through supportive care.

## Introduction

Diabetic amyotrophy, also known as diabetic lumbosacral radiculoplexus neuropathy, is an acute motor neuropathy that often presents with disabling pain and weakness [1,2]. It affects approximately 1% of patients with type 2 diabetes mellitus and demonstrates a male predominance [3]. Clinically, it presents as an asymmetric neuropathy involving proximal muscle groups and may persist for several months or even up to two years [4]. In contrast to more common diabetic neuropathies, which primarily involve sensory nerve degeneration due to metabolic and oxidative stress, diabetic amyotrophy is thought to arise from a localized inflammatory microvasculitis affecting the vasa nervorum. This process leads to ischemic injury of motor nerves, thereby distinguishing it both pathophysiologically and clinically from distal symmetric polyneuropathy [5]. Histopathologic studies have demonstrated ischemic nerve injury and epineurial microvasculitis, which further supports this mechanism [6,7]. Cerebrospinal fluid findings are typically acellular with elevated protein levels [8], but there remains no definitive diagnostic test for diabetic amyotrophy [8].

The typical presentation involves a male patient in his sixth decade with relatively well-controlled diabetes who develops acute, severe, unilateral pain in the thigh, hip, or lower back, followed by progressive weakness and atrophy of proximal muscles, including the quadriceps, hip adductors, and iliopsoas. This is often accompanied by diminished reflexes, weight loss, and autonomic symptoms [2,5,8]. However, atypical presentations without significant pain or with inconclusive electrodiagnostic findings have been increasingly recognized, further complicating diagnosis [3].

Approximately 75% of cases begin unilaterally before progressing to asymmetric or fully bilateral involvement [2]. Patients most commonly present with proximal lower extremity pain that is followed by weakness and muscle atrophy [1]. Additional associated features may include depression and emotional lability [8]. Symptoms typically worsen over the first six months before gradually improving thereafter, with most patients demonstrating partial or substantial recovery over one to three years [3,9]. Further postmortem studies have reinforced the role of microvasculitis and ischemic injury in the pathogenesis of diabetic lumbosacral radiculoplexus neuropathy [10]. This report presents an atypical case of proximal lower extremity weakness in a diabetic patient in whom the initial suspicion of diabetic amyotrophy was not supported by diagnostic testing. This case underscores the importance of a structured evaluation, careful diagnostic interpretation, and personalized patient management.

## Case presentation

An 80-year-old male with a history of type 2 diabetes mellitus, hypertension, prior stroke, and polycythemia vera presented with multiple falls over the course of one week due to progressive right lower extremity weakness and gait instability. He denied pain but reported continued distal lower extremity weakness. The patient reported the acute onset of right hip discomfort, balance instability, and dizziness with positional changes. His diabetes was suboptimally controlled, with a hemoglobin A1c of 7.6% (Table 1). His medication regimen included empagliflozin, glipizide, and sitagliptin, and insulin therapy was initiated during hospitalization. A neurological examination revealed proximal right lower extremity weakness with associated right thigh muscle atrophy, diminished right Achilles reflex, reduced bilateral patellar reflexes, and impaired balance and ambulation. The laboratory findings are summarized in Table 1.

**Table 1:** Laboratory findings Laboratory findings demonstrate elevated inflammatory markers and hyperglycemia with suboptimal glycemic control CRP: C-reactive protein; ESR: erythrocyte sedimentation rate; HbA1c: hemoglobin A1c

Laboratory test	Patient value	Reference range
CRP	2.9 mg/dL	<0.5 mg/dL
ESR	35 mm/hr	0–20 mm/hr
Glucose	135 mg/dL	70–99 mg/dL (fasting)
HbA1c	7.60%	<5.7% (normal)

Initial workup included a trial of carbidopa/levodopa without improvement, reducing the likelihood of Parkinsonian etiology. CT of the head and MRI of the brain were unremarkable. MRI of the lumbar spine and pelvic imaging did not reveal structural abnormalities such as disc herniation or stenosis. Orthostatic vital signs were within normal limits.

Nerve conduction studies (Tables 2-4) and electromyography (Table 5) demonstrated positive sharp waves in distal right lower extremity muscles with preserved conduction in proximal muscle groups. These findings suggested peripheral neuropathy with possible right-sided peroneal involvement rather than classic diabetic amyotrophy. The patient’s management included supportive care, physiotherapy, and continued diabetes monitoring and control.

**Table 2:** H-reflex studies H-reflex studies demonstrate absent responses in the bilateral tibial nerves

Nerve	Muscle	Result
Right tibial	Gastrocnemius	No response (NR)
Left tibial	Gastrocnemius	No response (NR)

**Table 3:** Antisensory nerve conduction studies Antisensory nerve conduction studies demonstrate absent sensory responses in the bilateral saphenous and sural nerves

Nerve	Site	Result
Left saphenous	Anterior medial malleolus	No response (NR)
Right saphenous	Anterior medial malleolus	No response (NR)
Left sural	Lateral malleolus	No response (NR)
Right sural	Lateral malleolus	No response (NR)

**Table 4:** Motor nerve conduction studies Motor nerve conduction studies demonstrate reduced amplitudes in the bilateral peroneal nerves and absent tibial motor responses

Nerve	Site	Onset (ms)	Normal onset (ms)	Amplitude (mV)	Normal Amp (mV)
Left peroneal (EDB)	Ankle	4.9	<6.1	0.8	>2.5
Right peroneal (EDB)	Ankle	5.5	<6.1	0.5	>2.5
Left tibial (AHB)	—	NR	—	—	—
Right tibial (AHB)	—	NR	—	—	—

**Table 5:** Electromyography (EMG) findings Electromyography demonstrates active denervation in distal right lower extremity muscles, including the extensor hallucis longus and anterior tibialis, with preserved proximal muscle function. These findings support a peripheral neuropathy with focal involvement

Side	Muscle	Nerve	Root	Fibrillations/PSWs	Amplitude	Duration	Recruitment
Right	Extensor hallucis longus	Deep peroneal	L5-S1	Present (+)	Increased	Increased	Normal
Right	Anterior tibialis	Deep peroneal	L4-5	Present (+)	Increased	Increased	Normal
Right	Gastrocnemius	Tibial	S1-2	Normal	Normal	Normal	Normal
Right	Vastus lateralis	Femoral	L2-4	Normal	Normal	Normal	Normal
Right	Adductor longus	Obturator	L2-4	Normal	Normal	Normal	Normal
Right	Biceps femoris	Sciatic	L5-S1	Normal	Normal	Normal	Normal
Left	Extensor hallucis longus	Deep peroneal	L5-S1	Normal	Normal	Normal	Normal
Left	Anterior tibialis	Deep peroneal	L4-5	Normal	Normal	Normal	Normal
Left	Gastrocnemius	Tibial	S1-2	Normal	Normal	Normal	Normal
Left	Vastus lateralis	Femoral	L2-4	Normal	Normal	Normal	Normal
Left	Adductor longus	Obturator	L2-4	Normal	Normal	Normal	Normal
Left	Biceps femoris	Sciatic	L5-S1	Normal	Normal	Normal	Normal

## Discussion

This case illustrates the diagnostic complexity of proximal lower extremity weakness in diabetic patients, particularly in the absence of classic features of diabetic amyotrophy. While diabetic amyotrophy is commonly associated with severe unilateral pain followed by weakness and atrophy, this patient presented with minimal pain and inconclusive electrodiagnostic findings, highlighting a less typical presentation that has been increasingly recognized in the literature [3].

Despite a clinical presentation suggestive of diabetic amyotrophy, the absence of hallmark diagnostic findings, including asymmetric denervation on electromyography, significant proximal nerve involvement, and characteristic pain, made this diagnosis less likely. Instead, electrodiagnostic evidence pointed toward a peripheral neuropathic process with possible focal radiculopathy, although imaging studies failed to identify structural causes such as disc herniation or spinal stenosis. This discordance between clinical presentation and diagnostic findings necessitated a diagnosis of exclusion and underscores the limitations of current diagnostic frameworks.

The absence of standardized diagnostic criteria for diabetic amyotrophy contributes to delayed diagnosis and variability in management [2,6]. Although electromyography and nerve conduction studies are central to evaluation, their findings may not always correlate with clinical suspicion, particularly in early or atypical cases [2,5]. Histopathologic and postmortem studies have demonstrated that microvasculitis and ischemic injury play a central role in disease pathogenesis. Yet, these processes are not readily captured through routine diagnostic testing, further complicating clinical evaluation [7-10]. In such scenarios, clinical judgment remains essential in guiding management and avoiding unnecessary or invasive interventions.

Even in the absence of a definitive diagnosis, early initiation of supportive care, including physical therapy, optimization of glycemic control, and fall-prevention strategies, may improve functional outcomes and quality of life [1,4]. Diabetic amyotrophy is typically a self-limiting condition, with most patients demonstrating partial or substantial recovery within one to three years [1,9]. However, delayed recognition or misclassification may prolong morbidity and negatively impact patient quality of life.

This case is notable for the absence of classic pain and inconclusive electrodiagnostic findings, highlighting a diagnostically challenging presentation that is not well represented in current literature. It further emphasizes the need for improved diagnostic frameworks that integrate clinical, electrodiagnostic, and emerging pathophysiologic insights to better guide early recognition and management of diabetic neuropathies.

## Conclusions

This report underscores the challenges in diagnosing diabetic amyotrophy, particularly in atypical presentations lacking classic clinical and electrodiagnostic features. The reliance on an exclusion-based diagnosis highlights the urgent need for standardized, evidence-based diagnostic criteria. A comprehensive assessment, including electrodiagnostics and imaging, is essential to guide accurate diagnosis and appropriate treatment, while improved frameworks may allow for earlier recognition, reduced diagnostic uncertainty, and more timely initiation of supportive therapies. Additionally, this report emphasizes the importance of patient-centered care, where management focuses not only on diagnostic clarity but also on functional recovery and quality of life. Although diagnostic certainty remains important, symptom-directed therapy and early rehabilitation can significantly improve outcomes, even when a precise etiology remains unclear. Continued reporting of atypical cases is essential to refine diagnostic approaches and enhance clinical understanding of the spectrum of diabetic neuropathies.
